# Ozone and Other Air Pollutants and the Risk of Oral Clefts

**DOI:** 10.1289/ehp.11311

**Published:** 2008-06-02

**Authors:** Bing-Fang Hwang, Jouni J.K. Jaakkola

**Affiliations:** 1 Department of Occupational Safety and Health, College of Public Health, China Medical University, Taichung, Taiwan; 2 Institute of Occupational and Environmental Medicine, The University of Birmingham, Birmingham, United Kingdom; 3 Institute of Health Sciences, University of Oulu, Oulu, Finland

**Keywords:** air pollution, cleft lip, ozone, traffic

## Abstract

**Background:**

Air pollution influences the development of oral clefts in animals. There are few epidemiologic data on the relation of prenatal air pollution exposure and the risk of oral clefts.

**Objectives:**

Our goal in this study was to assess the relations between exposure to ambient air pollution and the risk of cleft lip with or without cleft palate (CL/P).

**Methods:**

We conducted a population-based case–control study of all 653 cases of CL/P and a random sample of 6,530 control subjects from 721,289 Taiwanese newborns in 2001–2003. We used geographic information systems to form exposure parameters for sulfur dioxide, nitrogen oxides, ozone, carbon monoxide, and particulate matter with an aerodynamic diameter ≤ 10 μm (PM_10_) during the first 3 months of pregnancy using inverse distance weighting method. We present the effect estimates as odds ratios (ORs) per 10-ppb change for SO_2_, NO_x_, and O_3_, 100-ppb change for CO, and 10-μg/m^3^ change for PM_10_.

**Results:**

The risk of CL/P was increased in relation to O_3_ levels in the first gestational month [adjusted OR = 1.20; 95% confidence interval (CI), 1.02–1.39] and second gestational month (adjusted OR = 1.25; 95% CI, 1.03–1.52) in the range from 16.7 ppb to 45.1 ppb, but was not related to CO, NO_x_, SO_2_, or PM_10_.

**Conclusions:**

The study provides new evidence that exposure to outdoor air O_3_ during the first and second month of pregnancy may increase the risk of CL/P. Similar levels of O_3_ are encountered globally by large numbers of pregnant women.

The prevalence of oral clefts varies broadly from 0.06% to 0.17% in Caucasian births (Gorlin et al. 1990). Both genetic and environmental factors play important roles in the etiology of oral clefts (Zeiger and Beaty 2002), and there is probably also genetic susceptibility to the effects of environmental exposures. Ozone and carbon monoxide are toxic for the fetal development in rats and have been shown to produce skeletal malformation in animals (Garvey and Longo 1978; Kavlock et al. 1979; Longo 1977). Air pollution may influence the development of skeletal malformation through various biologic mechanisms, including hemodynamic, anoxic events, oxidative stress, and toxicity to certain cell populations during pregnancy (Ritz et al. 2002).

Two previous epidemiologic studies have elaborated the effects of exposure to ambient air pollution during pregnancy on the risk of birth defects (Gilboa et al. 2005; Ritz et al. 2002). In a case–control study in Southern California, Ritz et al. (2002) found an association between CO exposure during the second trimester and the risk of ventral septal defects and between second-month O_3_ exposure and the risk of aortic artery and valve defects, pulmonary artery and valve anomalies, and conotruncal defects. Gilboa et al. (2005) in a population-based case–control study in Texas found that the risk for aortic artery and valve defects, pulmonary artery and valve anomalies, and conotruncal defects was related to second-month O_3_ exposure. Both studies assessed also the relations between exposure to air pollutants and the risk of oral clefts, and reported weak positive, but statistically nonsignificant associations with O_3_ exposure.

We conducted a nationwide population-based case–control study in Taiwan to assess the effects of ambient air pollution exposure during pregnancy on the risk of cleft lip with or without palate (CL/P). We focused on predominantly traffic-related pollutants such as nitrogen oxides, CO, and O_3_ and air pollutants mainly from other fossil-fuel combustion sources, such as sulfur dioxide and particles with an aerodynamic diameter of ≤ 10 μm (PM_10_).

## Materials and Methods

### Study design

This was a population-based case–control study of CL/P. The source population consisted of all 721,289 births registered by the Taiwanese Birth Registry from 2001 through 2003. Our goal was to identify all the cases of CL/P in the source population during the study period. We randomly selected control subjects from the source population. The study was reviewed and approved by the Institutional Review Board of the College of Public Health, China Medical University.

### Definition and selection of cases

All births are compulsorily reported within 15 days to the Taiwan Local Household Registry, which is managed by the Taiwan Department of Health. Taiwanese pregnant women are almost all covered by national health insurance (>99%), and access to prenatal care is free and good (at least 10 times during pregnancy). The follow-up time is from 1 month after conception through 7 days after birth. Birth defects are diagnosed mostly by a physician, most often by a pediatrician using ultrasound. A validation study of the Taiwanese birth registration showed a low percentage of missing information (1.6%) and high degree of validity (sensitivity and specificity were 92.8% and 99.6%, respectively) and reliability (Cohen’s kappa measure of agreement was 0.92) for preterm births (< 37 weeks of gestational age) (Lin et al. 2004).

**Figure 1:**
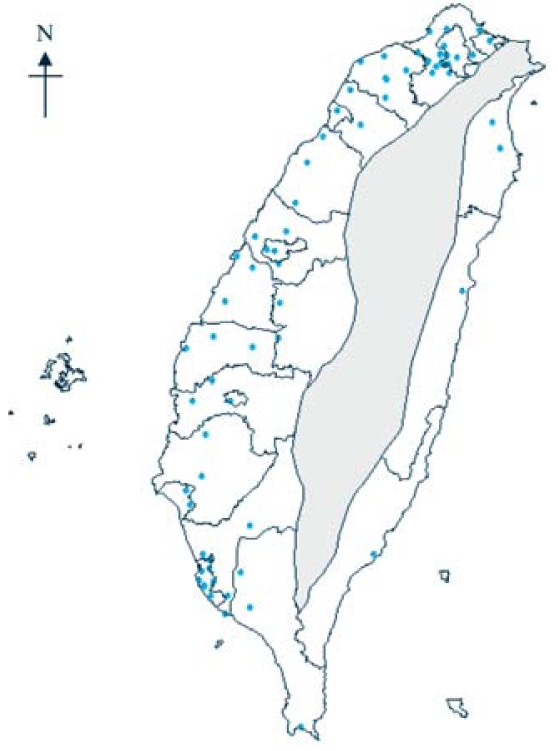
Geographic locations of 72 air pollution monitoring stations (shown in dots) in Taiwan. Shaded area indicates the mountain area that has no air monitoring stations.

We identified all births with CL/P from the Taiwanese Birth Registry from 2001 through 2003. The definition of CL/P was a fissure or elongated opening of the lip; cleft palate was a fissure in the roof of the mouth. We based the definition on the U.S. Committee of Federal and State Health Statistics Officials for the National Association of Public Health Statistics and Information Systems (Wyszynski and Wu 2002a). We identified a total of 653 subjects with sufficient information on gestational age and air pollutants and excluded 20 cases from the mountain area because of missing air pollution data (Figure 1).

### Selection of control subjects

We randomly drew the control subjects from the source population. The eligibility criteria included being born during the study period and having no birth defects, information on gestational age, and sufficient information on air pollutants. The case–control ratio was approximately 1:10 to approach optimal statistical power. The final study population included 6,530 controls.

### Exposure assessment

Complete monitoring data for the air pollutants SO_2_, NO_x_, O_3_, CO, and PM_10_, as well as daily temperature and relative humidity, are available from 1994 for 72 Taiwan Environmental Protection Agency (EPA) monitoring stations on Taiwan’s main island (Figure 1). Concentrations of each pollutant are measured continuously and reported hourly—CO by nondispersive infrared absorption, NO_x_ by chemiluminescence, O_3_ by ultraviolet absorption, SO_2_ by ultraviolet fluorescence, and PM_10_ by beta gauge.

We identified the map coordinates of the monitoring stations and air pollution sources. We managed the data by the Arcview 3.2 (ESRI, Redlands, CA, USA) geographic information system (GIS). We integrated the air pollutant measurements from the Taiwan EPA monitoring stations into monthly point data. We interpolated these data to pollutant surfaces using the inverse distance weighting method, which is the simplest interpolation method: Users identify a neighborhood about the interpolated point and take a weighted average of the observed values within this neighborhood. The weights are a decreasing function of distance. The user has control over the mathematical form of the weighting function and the size of the neighborhood (expressed as a radius or a number of points) (Fisher et al. 1987). The weighting function [*w*(*p*)] is *w*(*d*) = 1/*d* *^p^* with *p* > 0, where *d* represents distance away from the specified monitoring station. The value of *p* is specified by the user. The most common choice is *p* = 2. The air pollutant surfaces derived as described below provide the spatial distribution of each pollutant. We extracted the air pollutant information for each woman during pregnancy, corresponding to the center of townships or districts, from the derived concentration surface maps using ArcGIS Spatial Analyst tool (developed by ESRI) (e.g., center of a polygon). We excluded 25 of 365 townships located in the mountain area where there are no air monitoring stations (Figure 1). This represents only 2% of all the births.

We calculated exposure parameters from the monthly average concentrations for the duration of pregnancies from 2000 through 2003. Based on the date of birth and gestational age, we estimated the monthly average concentration corresponding to the first, second, and third month of gestation.

### Covariates

We used routine birth registry data to construct the following covariates: sex of infant (male, female), maternal age (< 20 years, 20–34 years, ≥ 35 years), plurality (singleton, multiple birth), gestational age (< 37 weeks, ≥ 37 weeks), and season of conception (spring, summer, fall, winter). We received municipal-level data from the Department of Household Registration Affairs, Taiwanese Population Data Services, which we used to construct municipal-level population density, which is a measure of the proportion of urban population in the municipality.

### Statistical methods

We focused on the first 3 months of pregnancy, because the relevant embryologic period for oral cleft is from the 4th to the 12th week of gestation (Wyszynski and Wu 2002b). We used odds ratio (OR) as a measure of the relation between exposure to air pollution and the risk of CL/P. We estimated adjusted ORs using logistic regression analysis and present the results from the models as ORs, along with their 95% confidence intervals (CIs). We assessed the goodness of fit with likelihood ratio tests to determine whether a variable contributed significantly to the model. First, we fitted a full model with a complete set of covariates. To elaborate sources of confounding, we fitted models with different combinations of covariates and compared the effect from models with and without the covariate of interest. If the inclusion of a covariate changed the studied effect estimate more than 10%, we kept the corresponding covariate in the final model (Gilboa et al. 2005; Greenland 1989; Ritz et al. 2007). We first fitted one-pollutant models and then considered two-pollutant models by fitting one traffic-related and one stationary fossil-fuel combustion-related pollutant. Finally, we fitted two-pollutant models with O_3_ and another pollutant. The two-pollutant models provide estimates of the independent effects of CO, NO_x_, SO_2_, PM_10_, and O_3_ on CL/P controlling for the second pollutant in the model. We also considered three-pollutant models with one traffic-related pollutant, one stationary fossil-fuel combustion-related pollutant, and O_3_. We present the effect of each pollutant on the risk of CL/P as ORs per 10-ppb change for SO_2_, NO_x_, and O_3_, 100-ppb (10-pphm) change for CO, and 10-μg/m^3^ change for PM_10_, along with their 95% CIs.

## Results

### Characteristics of control and case subjects

A larger proportion of cases than controls was male (χ^2^ = 3.8, *p* = 0.05) and had older mothers (χ^2^ = 5.8, *p* = 0.06) and shorter gestational age (< 37 weeks) (χ^2^ = 306, *p* = 0.001) (Table 1). We adjusted for these factors in the multivariate analysis.

### Air pollution

Table 2 presents the distributions of the monthly mean air pollutant concentrations during the first 3 months pregnancy. The correlation between NO_x_ and CO trimester average concentrations during the first trimester was high (*r* = 0.82), which reflects the common source of motor vehicles. The concentrations of PM_10_ and SO_2_ were also highly correlated (*r* = 0.50), indicating a common source of stationary fuel combustion, although SO_2_ concentrations were also correlated with both traffic-related pollutants. The concentration of O_3_ was negatively correlated with the mainly traffic-related pollutants, but positively with PM_10_ and SO_2_, and it was only weakly correlated with that of traffic-related and stationary fossil-fuel combustion-related air pollutants (Table 3).

### Air pollution and the risk of CL/P

Table 4 shows the effect estimates from one-pollutant and three-pollutant models. Table 5 displays the results from the two-pollutant models. In the one-pollutant model, the risk of CL/P was related to O_3_ levels, particularly in the first month of pregnancy (adjusted OR = 1.17 per 10-ppb change; 95% CI, 1.01–1.36) and second month of pregnancy (adjusted OR = 1.22; 95% CI, 1.03–1.46). The effect estimate for the third-month exposure to O_3_ was slightly elevated but not statistically significant (adjusted OR = 1.09; 95% CI, 0.93–1.26) (Table 4). In the three-pollutant models, the effect estimates for O_3_ exposure were stable for the four different combinations of pollutants, varying between 1.18 and 1.20 for the first month and between 1.21 and 1.25 for the second month, and were all statistically significant (Table 4). The adjusted OR for a 100-ppb change in CO was 1.01 (95% CI, 0.97–1.04) for the first month of pregnancy, and the estimates changed little when we added a second or third pollutant. The adjusted OR for a 10-ppb change in SO_2_ alone was 0.92 (95% CI, 0.63–1.35) for the first month, but including both of the traffic-related pollutants and O_3_ reduced the effect estimate substantially. The risk of CL/P was not related to traffic-related (NO_x_) and stationary fossil-fuel combustion-related (PM_10_) air pollutant concentration.

In summary, we found positive statistically significant associations for first- and second-month O_3_ exposure. In contrast, we found negative or weak associations for traffic-related (CO and NO_x_) and stationary fossil-fuel combustion-related (SO_2_ and PM_10_) pollutants.

## Discussion

The risk of CL/P increased with increasing O_3_ levels during the first and second month of pregnancy. The effect estimate indicating an approximately 20% risk increase per 10-ppb increase in O_3_ level was stable with different combinations of air pollutants in the multi-pollutant models. The risk of CL/P was not related to two traffic-related pollutants (NO_x_ and CO) or two stationary fossil-fuel combustion-related pollutants (PM_10_ and SO_2_). The results provide evidence that O_3_ exposure in the most susceptible time periods in pregnancy may increase the risk of CL/P. This finding is consistent with animal toxicologic evidence of the effects of O_3_ (Kavlock et al. 1979; Lohnes et al. 1995; Takahashi et al. 1990).

### Validity of results

We were able to include a high proportion of Taiwanese CL/P cases (98%) because all births are compulsorily reported to the Taiwan Local Household Registry within 15 days. Thus, the magnitude of potential selection bias was likely to be negligible. Important features in the Taiwan national health care system limit the amount of outcome misclassification. Taiwanese pregnant women are almost all covered by health insurance (> 99%), and access to prenatal care is free of charge (at least 10 times during pregnancy). The follow-up time is from 1 month after conception through 7 days after birth. In our study, the cases had a higher proportion of premature infants than did the controls. Although we included gestational age (< 37 weeks vs. ≥ 37 weeks) in the multivariate analysis adjusting for the potential difference between cases and controls, we still cannot rule out the possibility that the presence of premature infants in the case roup may augur other exposures that also may mediate risk for CL/P.

We were able to adjust for several confounders in logistic regression analysis to eliminate these factors as a potential explanation for our results. Although there is evidence that oral clefts are related to maternal smoking, folic acid deficiency, and genetic factors (Shaw et al. 2005; Zeiger et al. 2005), we have no reason to suspect that these factors would be associated with exposure. Information on maternal smoking was not available for our study, but from other sources (Chen et al. 2002) we know that the prevalence of smoking during pregnancy is low (4.9%), and therefore the magnitude of potential confounding is small. We based our outcome assessment on a physician’s diagnosis, usually a pediatrician, within 15 days of the delivery. CL/P is present and relatively easily detectable after delivery. Regional variation in diagnostic practice and reporting was a possible source of misclassification, which may be related to exposure levels, because we based the exposure contrasts on regional differences. Adjustment for population density not only adjusted indirectly for municipal differences in these behavior factors, but also reduced any bias introduced by regional differences in diagnosis and reporting. However, residual confounding is still possible by unmeasured or poorly characterized factors or by other environmental toxicants. We systematically carried out stratified analyses in different categories of exposure and other covariates to elaborate the potential effect modification. The stratified analyses did not indicate any major effect modification.

Any known or unknown factors, such as physical activity, time spent outdoors, occupational status, air exchange, penetration, deposition, and emission strengths for indoor pollutants, could be responsible for the observed association between personal exposure and municipal level exposure. We assumed these errors to be nondifferential with respect to cases and controls. Therefore, such misclassification would lead to underestimation of the effect estimates. This was a common problem in all the previous studies assessing the effects of air pollution on the risk of pregnancy outcomes (Š rám et al. 2005).

A major challenge of this study was the imprecision of exposure assessment that we based on monthly municipal level air pollutant information. Navidi and Lurmann (1995) reported that when using the municipal level exposure obtained from air pollution monitoring stations as a proxy for personal exposure, the effect estimates are in general smaller than those based on personal assessment of exposure. A plausible mechanism of information bias is that pregnant women may change residential area, which will lead to exposure misclassification. Any random migration between cases and controls might introduce nondifferential misclassification and decrease the accuracy of exposure assessment. This most likely underestimates the air pollution effects rather than introducing a positive bias in the associations.

In general, the assessment of the independent effects of different pollutants is difficult, because urban air pollution constitutes a complex mixture of several compounds. Typically, NO_x_ and CO concentrations are highly correlated (*r* = 0.82) because they both are predominantly from vehicle emissions. Similarly, PM_10_ and SO_2_ are somewhat correlated (*r* = 0.50), having stationary fossil combustion processes as important sources. In addition, PM_10_ may also be partly traffic related, because it is correlated with NO_x_ (*r* = 0.56). O_3_ is, as a product of photochemical oxidation, a secondary air pollutant generated in the troposphere from precursors of the vehicle emissions (nitrogen dioxide and hydrogen carbon), but the concentrations of O_3_ are slightly negatively related to NO_x_ (*r* = −0.05) and CO (*r* = −0.19) concentrations. This enables somewhat more valid assessment of the effects of O_3_ independent from other traffic-related pollutants. In the modeling, we were able to control for one stationary fossil-fuel pollutant at a time as a potential confounder when assessing the effect of one of the traffic-related pollutants and vice versa.

### Synthesis with previous knowledge

In the present study, we found a 20% increase in the risk of CL/P per 10-ppb increase in O_3_ exposure during the second month of exposure. The average monthly means of O_3_ varied from 16.7 ppb to 45.1 ppb. Two previous population-based case–control studies, conducted in Southern California (Ritz et al. 2002) and in Texas (Gilboa et al. 2005), have elaborated the relations between exposure to ambient air pollution and the risk of oral clefts. Both studies reported elevated but not statistically significant effect estimates for O_3_ exposure, which is in line with our results in a similar range of O_3_ levels. The Southern California study, with 450 CL/P cases and 3,000 controls, reported an adjusted OR of 1.13 per 10 ppb (95% CI, 0.90–1.40) during the second month; the range of exposure was 1.4–99.4 ppb (Ritz et al. 2002). The Texas study of 305 CL/P cases and 3,594 controls reported an adjusted OR of 1.09 (95% CI, 0.70–1.69) for the fourth quartile (≥ 31 ppb) contrasted with the first quartile (< 18 ppb) of exposure during 3–8 weeks of pregnancy (Gilboa et al. 2005).

The evidence of a positive association between the risk of CL/P and exposure to O_3_, provided by our study, is compatible with toxicologic studies (Kavlock et al. 1979; Lohnes et al. 1995; Takahashi et al. 1990). Kavlock et al. (1979) reported that high exposure to O_3_ (> 1.26 ppm) during organogenesis reduced skeletal ossification. In rats, exposure to 0.4 ppm O_3_ for 1–4 days lowered the serum retinol concentration by 85% (Takahashi et al. 1990), which supports the hypothesized adverse effects of O_3_, because vitamin A deprivation during organogenesis is known to cause several congenital defects (Lohnes et al. 1995). The risk of CL/P was associated with the levels of O_3_. The most susceptible time periods in pregnancy for the effects of O_3_ were the first and second month of gestation. O_3_ is a secondary pollutant in the atmosphere produced from traffic exhausts but scavenged by direct motor vehicle emissions. O_3_ is a known strong oxidizing agent that can generate hydrogen peroxide, hydroxyl radicals, and superoxides. It could contribute to oxidative stress and causally influence the development of oral clefts.

Our finding of a lack of association between the risk of CL/P and traffic-related (CO, NO_x_) and combustion-related (SO_2_, PM_10_) air pollutant levels is consistent with the results from two previous studies in Southern California and Texas (Gilboa et al. 2005; Ritz et al. 2002). The present study provides an original finding that the effect of exposure to outdoor air O_3_ during the first and second month of pregnancy increases the risk of CL/P. Given that similar levels are encountered globally by large numbers of pregnant women, O_3_ may be an important determinant of orofacial birth defects.

## Figures and Tables

**Table 1:** Characteristics of controls and cases of cleft lip in Taiwan, 2001–2003 [no. (%)].

Characteristic	Controls	Cases	Total
Total	6,530 (100)	653 (100)	7,183 (100)
Sex of infant	χ^2^ (df = 1) = 3.8, *p*-value = 0.05		
Male	3,429 (52.5)	369 (56.5)	3,798 (52.9)
Female	3,101 (47.5)	284 (43.5)	3,385 (47.1)
Maternal age (years)	χ^2^ (df = 2) = 5.8, *p*-value = 0.06		
< 20	272 (4.2)	35 (5.4)	307 (4.3)
20–34	5,693 (87.2)	548 (83.9)	6,241 (86.9)
≥ 35	565 ( (8.6)	70 (10.7)	635 (8.8)
Plurality	χ^2^ (df = 1) = 0.1, *p*-value = 0.75		
Singleton	6,373 (97.6)	636 (97.4)	7,009 (97.6)
Multiple birth	157 (2.4)	17 (2.6)	174 (2.4)
Gestational age	χ^2^ (df = 1) = 306, *p*-value = 0.001		
< 37 weeks	543 (8.3)	197 (30.2)	740 (10.3)
≥ 37 weeks	5,987 (91.7)	456 (69.8)	6,443 (89.7)
Population density (people/km^2^)	χ^2^ (df = 2) = 1.9, *p*-value = 0.37		
< 1,000	1,597 (24.4)	176 (27.0)	1,773 (24.7)
1,000–5,000	2,793 (42.8)	270 (41.3)	3,063 (42.6)
> 5,000	2,140 (32.8)	207 (31.7)	2,347 (32.7)
Season of conception	χ^2^ (df = 3) = 2.8, *p*-value = 0.42		
Spring	1,493 (22.9)	144 (22.1)	1,637 (22.8)
Summer	1,620 (24.5)	176 (27.0)	1,778 (24.8)
Fall	1,573 (24.1)	143 (21.9)	1,716 (23.9)
Winter	1,862 (28.5)	190 (29.1)	2,052 (28.6)

**Table 2:** Mean and distribution of air pollutants in different seasons from 72 monitoring stations in Taiwan, 2001–2003.

Pollutant	Mean ± SD	Minimum	25th percentile	Median	75^th^ percentile	Interquartile range^a^	Maximum
O_3_ (ppb)							
Spring	29.69 ± 6.06	16.30	25.47	28.90	32.97	7.50	50.27
Summer	23.56 ± 4.14	14.37	20.90	23.57	25.37	4.47	39.10
Fall	29.07 ± 5.42	15.87	25.80	28.93	32.07	6.27	42.80
Winter	26.92 ± 5.57	17.60	22.80	26.27	30.83	8.03	48.13
Average	27.31 ± 4.88	16.70	24.43	26.78	30.11	5.68	45.08
CO (pphm)							
Spring	71 ± 37	27	51	64	80	29	247
Summer	60 ± 40	23	41	50	66	25	256
Fall	65 ± 42	26	46	57	71	25	306
Winter	78 ± 42	25	53	71	88	35	298
Average	69 ± 40	25	48	62	76	28	277
NO_x_ (ppb)							
Spring	21.97 ± 8.17	1.23	17.43	21.72	26.27	8.84	49.68
Summer	15.89 ± 6.86	0.95	11.54	14.47	19.08	7.54	41.58
Fall	17.60 ± 6.53	0.83	13.68	17.49	21.25	7.57	39.93
Winter	25.33 ± 8.88	1.08	19.23	25.70	30.87	11.64	48.38
Average	20.20 ± 7.24	1.02	16.03	20.16	23.94	7.91	44.20
SO_2_ (ppb)							
Spring	4.22 ± 2.59	0.23	2.53	3.67	5.18	2.64	12.63
Summer	3.35 ± 2.00	0.20	1.96	2.98	4.23	2.27	10.02
Fall	3.41 ± 2.07	0.20	2.00	2.83	4.54	2.54	11.07
Winter	4.88 ± 3.71	0.20	2.96	3.95	5.38	2.42	17.93
Average	3.96 ± 2.36	0.21	2.36	3.42	5.01	2.65	11.48
PM_10_ (μg/m^3^)							
Spring	64.44 ± 16.21	23.33	53.00	67.00	75.42	22.42	94.33
Summer	39.11 ± 8.31	17.33	33.92	39.67	43.42	9.50	60.00
Fall	47.76 ± 11.77	21.00	39.33	49.17	55.66	16.33	72.00
Winter	68.00 ± 21.88	21.33	50.25	67.17	84.67	34.42	116.0
Average	54.83 ± 13.07	20.75	44.81	57.17	64.54	19.73	78.05

a Range from 25th to 75th percentile of site-specific concentrations.

**Table 3:** Correlations of air pollutants’ trimester average concentration during the first 3 months pregnancy.

	CO	NO_x_	O_3_	PM_10_	SO_2_
CO	1.00	0.82*	−0.19	−0.19	0.24
NO_x_		1.00	−0.05	0.56*	0.45*
O_3_			1.00	0.39	0.23
PM_10_				1.00	0.50*
SO_2_					1.00

* *p* < 0.05.

**Table 4:** Adjusted ORs^a^ (95% CIs) for cleft lip during the first 3 months of pregnancy in single- and three-pollutant models.

Pollutant	Single-pollutant model	Three-pollutant model 1 (O_3_ + CO + SO_2_)	Three-pollutant model 2 (O_3_ + NO_x_ + SO_2_)	Three-pollutant model 3 (O_3_ + CO + PM_10_)	Three-pollutant model 4 (O_3_ + NO_x_ + PM_10_)
O_3_ (10 ppb)					
1st month	1.17 (1.01–1.36)	1.20 (1.02–1.40)	1.20 (1.02–1.39)	1.20 (1.01–1.41)	1.18 (1.00–1.39)
2nd month	1.22 (1.03–1.46)	1.21 (1.03–1.42)	1.28 (1.06–1.56)	1.25 (1.04–1.51)	1.25 (1.03–1.52)
3rd month	1.09 (0.93–1.26)	1.12 (0.96–1.31)	1.12 (0.96–1.31)	1.11 (0.94–1.30)	1.09 (0.92–1.29)
CO (100 ppb)					
1st month	1.00 (0.96–1.04)	1.01 (0.97–1.04)		1.01 (0.97–1.05)	
2nd month	1.00 (0.96–1.03)	1.00 (0.97–1.04)		1.01 (0.97–1.05)	
3rd month	1.00 (0.96–1.03)	1.00 (0.97–1.04)		1.00 (0.96–1.04)	
NO_x_ (10 ppb)					
1st month	0.95 (0.81–1.12)		0.97 (0.81–1.15)		0.95 (0.78–1.15)
2nd month	0.96 (0.81–1.13)		1.06 (0.88–1.28)		1.03 (0.84–1.26)
3rd month	0.93 (0.79–1.09)		0.99 (0.83–1.18)		0.95 (0.78–1.15)
SO_2_ (10 ppb)					
1st month	0.92 (0.63–1.35)	0.82 (0.55–1.22)	0.85 (0.55–1.31)		
2nd month	0.84 (0.57–1.25)	0.74 (0.49–1.12)	0.70 (0.44–1.11)		
3rd month	0.72 (0.47–1.08)	0.67 (0.44–1.03)	0.68 (0.43–1.07)		
PM_10_ (10 μg/m^3^)					
1st month	1.01 (0.96–1.06)			0.99 (0.94–1.04)	1.00 (0.94–1.06)
2nd month	1.00 (0.95–1.05)			0.99 (0.94–1.04)	0.98 (0.92–1.05)
3rd month	0.99 (0.95–1.05)			0.98 (0.93–1.04)	1.00 (0.93–1.06)

a Logistic regression analysis adjusting for maternal age, plurality, gestational age, population density, and season of conception.

**Table 5:** Adjusted ORs^a^ (95% CIs) of CL/P exposure to first 3 months of pregnancy in two-pollutant models.

Pollutant	Two-pollutant model 1 (O_3_ + CO)	Two-pollutant model 2 (O_3_ + NO_x_)	Two-pollutant model 3 (O_3_ + SO_2_)	Two- pollutant model 4 (O_3_ + PM_10_)	Two- pollutant model 5 (CO + PM_10_)	Two- pollutant model 6 (CO + SO_2_)	Two- pollutant model 7 (NO_x_ + PM_10_)	Two- pollutant model 8 (NO_x_ + SO_2_)
O_3_ (10 ppb)								
1^st^ month	1.17 (1.01–1.37)	1.17 (1.01–1.37)	1.19 (1.02–1.40)	1.19 (1.01–1.40)				
2nd month	1.23 (1.03–1.48)	1.22 (1.02–1.46)	1.26 (1.05–1.51)	1.24 (1.03–1.48)				
3^rd^ month	1.09 (0.93–1.26)	1.08 (1.93–1.26)	1.12 (0.96–1.31)	1.10 (0.95–1.55)				
CO (100 ppb)								
1^st^ month	1.00 (0.97–1.04)				1.00 (0.96–1.04)	1.00 (0.97–1.04)		
2nd month	1.01 (0.97–1.05)				1.00 (0.96–1.04)	1.00 (0.96–1.04)		
3^rd^ month	1.00 (0.96–1.04)				1.00 (0.96–1.03)	1.00 (0.96–1.04)		
NO_x_ (10 ppb)								
1^st^ month		0.94 (0.80–1.11)					0.91 (0.75–1.10)	0.96 (0.80–1.14)
2nd month		0.99 (0.84–1.18)					0.94 (0.78–1.14)	0.98 (0.82–1.17)
3rd month		0.93 (0.79–1.10)					0.92 (0.76–1.11)	0.97 (0.81–1.15)
SO_2_ (10 ppb)								
1st month			0.82 (0.55–1.23)			0.92 (0.62–1.35)		0.96 (0.63–1.45)
2nd month			0.75 (0.50–1.13)			0.84 (0.57–1.25)		0.86 (0.56–1.31)
3rd month			0.67 (0.44–1.03)			0.72 (0.48–1.08)		0.74 (0.48–1.14)
PM_10_ (10 μg/m^3^)								
1st month				0.99 (0.94–1.04)	1.01 (0.96–1.06)		1.02 (0.97–1.08)	
2nd month				0.99 (0.94–1.04)	1.00 (0.95–0.1.05)		1.01 (0.95–1.07)	
3rd month				0.98 (0.93–1.04)	0.99 (0.95–1.05)		1.01 (0.95–1.07)	

a Logistic regression analysis adjusting for maternal age, plurality, gestational age, population density, and season of conception.
